# Role of E-type prostaglandin receptor EP3 in the vasoconstrictor activity evoked by prostacyclin in thromboxane-prostanoid receptor deficient mice

**DOI:** 10.1038/srep42167

**Published:** 2017-02-06

**Authors:** Zhenhua Li, Yingzhan Zhang, Bin Liu, Wenhong Luo, Hui Li, Yingbi Zhou

**Affiliations:** 1 Dept of Pathology, The 2nd Affiliated Hospital, Shantou University Medical College, Shantou, China; 2 Cardiovascular Research Center, Shantou University Medical College, Shantou, China; 3 The Central Lab, Shantou University Medical College, Shantou, China

## Abstract

Prostacyclin, also termed as prostaglandin I_2_ (PGI_2_), evokes contraction in vessels with limited expression of the prostacyclin receptor. Although the thromboxane-prostanoid receptor (TP) is proposed to mediate such a response of PGI_2_, other unknown receptor(s) might also be involved. TP knockout (TP^−/−^) mice were thus designed and used to test the hypothesis. Vessels, which normally show contraction to PGI_2_, were isolated for functional and biochemical analyses. Here, we showed that the contractile response evoked by PGI_2_ was indeed only partially abolished in the abdominal aorta of TP^−/−^ mice. Interestingly, further antagonizing the E-type prostaglandin receptor EP3 removed the remaining contractile activity, resulting in relaxation evoked by PGI_2_ in such vessels of TP^−/−^ mice. These results suggest that EP3 along with TP contributes to vasoconstrictor responses evoked by PGI_2_, and hence imply a novel mechanism for endothelial cyclooxygenase metabolites (which consist mainly of PGI_2_) in regulating vascular functions.

Cyclooxygenase (COX), which exists mainly as COX-1 and -2 isoforms, mediates the metabolism of arachidonic acid (AA) to produce vasoactive prostanoids^1^,^2^,^3^,^4^. Among them, thromboxane (Tx) A_2_ and prostacyclin (prostaglandin I_2_; PGI_2_) have been considered to represent two opposing regulatory mechanisms in the cardiovascular system. TxA_2_ is mainly produced in platelets and it acts on the thromboxane-prostanoid receptor (TP) to mediate vasoconstriction and platelet-aggregation. In contrast, PGI_2_ is mainly synthesized in the vascular endothelium and is proposed to activate the PGI_2_ receptor (IP) that mediates vasodilatation and opposes the effects of TP. An imbalance between the effects derived from endothelial PGI_2_ and those of platelet-produced TxA_2_ is though to result in the development of cardiovascular disorders, such as hypertension^1^,^2^,^3^,^4^,^5^,^6^.

On the other hand, in some vascular beds (including certain human vessels), PGI_2_ or endothelial COX metabolites (which consist mainly of PGI_2_) evoke contraction via the activation of TP^7^,^8^,^9^,^10^,^11^,^12^,^13^,^14^,^15^,^16^,^17^,^18^,^19^,^20^,^21^,^22^,^23^,^24^. Studies have further revealed that vasomotor reactions to PGI_2_ are modulated by both IP and TP; hence a vasoconstrictor response evoked by PGI_2_ or endothelial COX metabolites reflects limited expression or function of IP, which leads to the uncovering of vasoconstrictor activity derived from concurrently activated TP^8^,^20^,^21^,^22^,^25^,^26^,^27^,^28^,^29^,^30^,^31^,^32^. However, in some vessels, such as mouse abdominal aorta where IP is expressed (although to a lesser extent as compared to vessels showing dilation to the agonist), PGI_2_ does not evoke relaxation even after TP blockade^28^,^30^. Also, in some vascular beds, the contraction evoked by PGI_2_ or endothelial COX metabolites is less sensitive to TP blockade^11^,^22^. We propose that in addition to TP, other receptor(s) can also be involved in PGI_2_-evoked vasoconstrictor activity. However, the existence of such a mechanism or the identity of the additional receptor(s) remains to be elucidated. In addition, the involvement of TP in the vasoconstrictor activity of PGI_2_ has been primarily based on results with pharmacological blockade, which also inhibits contractions evoked by other PGs or AA-related metabolites^8^,^33^,^34^. Thus, it would also be of interest to evaluate the precise role of TP in PGI_2_-evoked vasoconstrictor responses with genetic manipulation.

To resolve the above issues, in this study we generated a strain of TP^−/−^ mice on a C57BL/6 background. Aortas, carotid and/or renal arteries, where PGI_2_ evokes vasoconstrictor response under normal conditions^26^,^28^,^30^,^35^, were isolated for biochemical and/or functional analyses.

## Results

### Mutation in TP^−/−^ mice and phenotype

**Figure 1:**
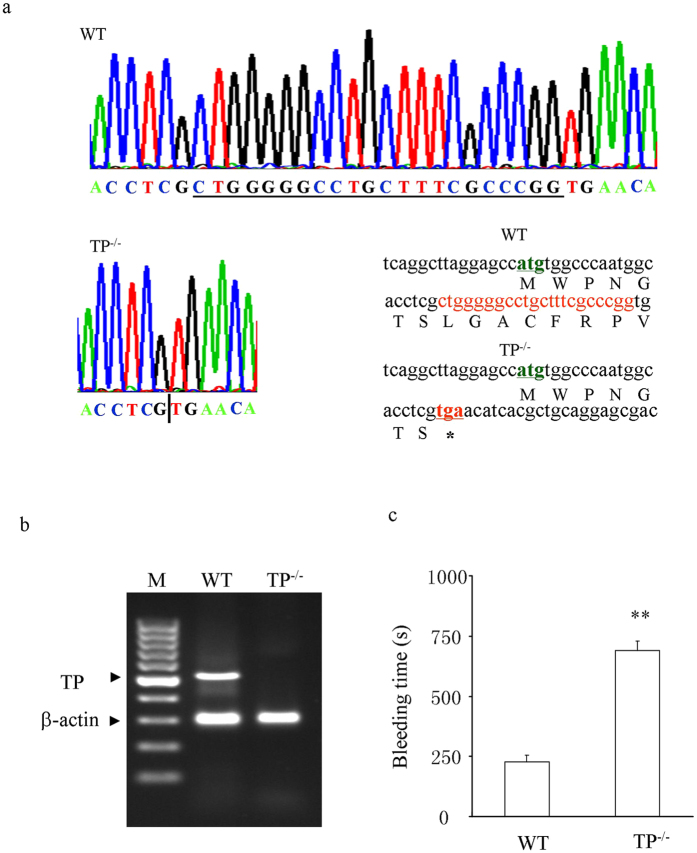
Mutation in TP^−/−^ mice and phenotype. (**a**) mutation in the TP locus. Top: WT DNA sequencing showing the surrounding sequences and the fragment to be deleted (underlined) in TP^−/−^ mice. Bottom left: sequencing of mutated DNA showing the deletion of 22 bp DNA fragment in TP^−/−^ mice. The bar separates the upper and down stream sequences of the deleted fragment. Bottom right: partial sequences of TP mRNA transcripts or those of proteins to be translated in WT (upper) and TP^−/−^ mice (lower). (**b**) RT-PCR showing the expressions of un-mutated mRNAs in WT and TP^−/−^ mouse aortas. Bands were visualized with a SYBR Safe DNA gel stain (Thermo Scientific) and the image was captured by an electrophoresis imaging cabinet (Universal Hood II; Bio-rad, Hercules, CA, USA). M: 100 bp ladder size marker (Thermo Scientific). (**c**) bleeding time in TP^−/−^ and WT mice. Values are expressed as mean ± SEM; n = 5; **P < 0.01.

As shown in Fig. 1a, sequencing of TP DNA PCR products revealed that as compared with that of wild-type (WT) mice, exon 3 of the TP locus in TP^−/−^ mice has a 22 bp fragment deletion (CTG GGG GCC TGC TTT CGC CCG G) in the coding area, which was 18 bp after the start codon (NCBI Reference Sequence: NM_009325.3). This resulted in a frame-shift in TP mRNA transcript and a premature termination of protein translation (only 7 amino acids were coded before the appearance of a stop codon (TGA) in TP^−/−^ mice; Fig. 1a, bottom right). Indeed, RT-PCR revealed that un-mutated TP mRNAs, which were abundant in WT aortas, were not detected in the TP^−/−^ counterparts (Fig. 1b). Also, compared to WT controls, TP^−/−^ mice had an elongated bleeding time (Fig. 1c). However, these mice appear normal, and show no overt abnormality in mean arterial blood pressure (MAP; 92.3 ± 3.3 vs. 95.0 ± 2.8 mmHg in WT mice, n = 5 for each; P > 0.05) or in reproduction.

### Effect of TP^−/−^ on contractions evoked by PGI_2_ and other prostanoids

**Figure 2:**
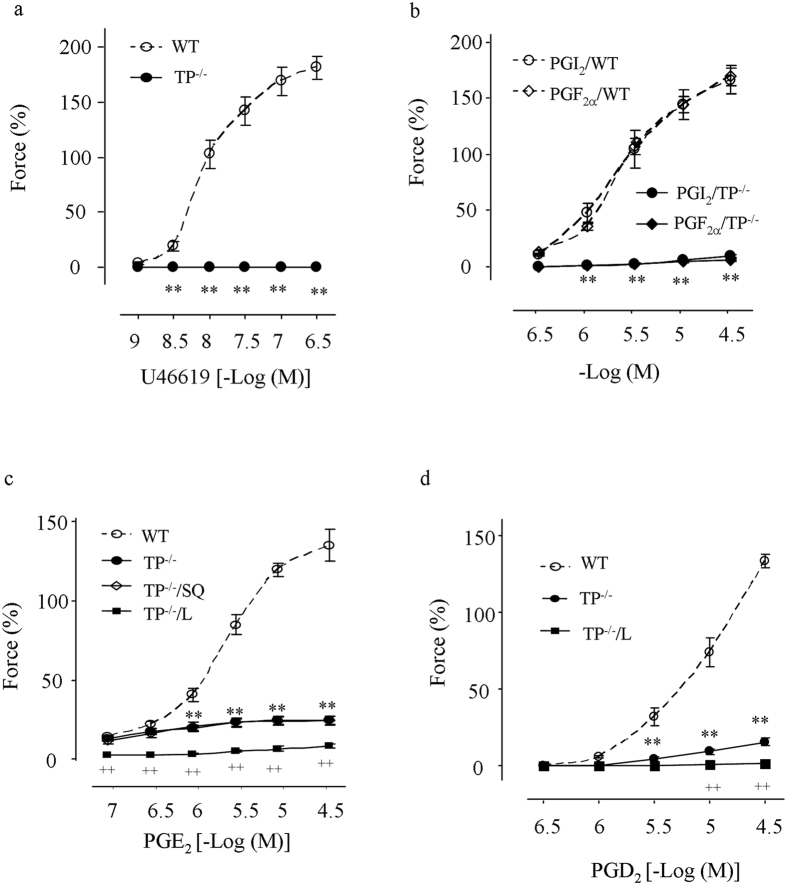
Responses to U46619 and PGs in L-NAME-treated WT and TP^−/−^ abdominal aortas. (**a,b**) comparison of contractions evoked by the TP agonist U46619 (**a**), and PGI_2_ or PGF_2α_ (**b**) in WT and TP^−/−^ vessels. (**c,d**) contraction to PGE_2_ (**c**) or PGD_2_ (**d**) in WT or TP^−/−^ mice and that of TP^−/−^ vessels treated with the TP antagonist SQ29548 (10 μM; +SQ) or the EP3 antagonist L798106 (1 μM; +L). Values are expressed as mean ± SEM; n = 5 for each. **P < 0.01 vs. the value of WT mice; ^++^*P* < 0.01 vs. TP^−/−^ mice.

Abdominal aortas were then isolated for functional analyses. Vessels were treated with the NO synthase (NOS) inhibitor N^ω^-nitro-L-arginine methyl ester (L-NAME; 1 mM). In WT vessels, the TP agonist U46619 evoked potent contraction as noted previously^28^; however, in TP^−/−^ mice, U46619 did not evoke any response (Fig. 2a). Interestingly, not only the contraction evoked by PGI_2_ (Fig. 2b), but that to PGF_2α_ (Fig. 2b), PGE_2_ (Fig. 2c), or PGD_2_ (Fig. 2d) was also diminished or largely removed in TP^−/−^ vessels. At the same time, the contraction to low concentrations (0.1–0.3 μM) of PGE_2_ remained intact in TP^−/−^ vessels, and this contraction was abolished by the E-type prostaglandin receptor EP3 antagonist L798106 (1 μM), but not by the TP antagonist SQ29548 (10 μM; Fig. 2c). In addition, L798106 abolished the remaining contraction evoked by PGD_2_ in TP^−/−^ vessels (Fig. 2d).

### PGI_2_-induced response in TP^−/−^ abdominal aortas precontracted with PE

**Figure 3:**
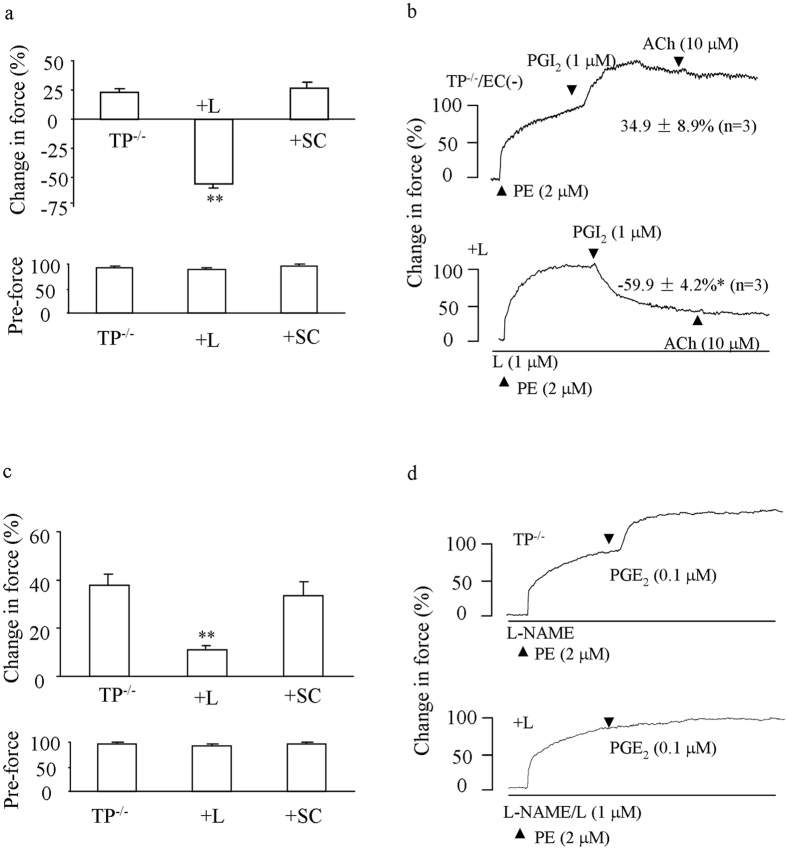
Effect of EP3 antagonism on the response to PGI_2_ or PGE_2_ in PE-pre-contracted TP^−/−^ abdominal aortas. (**a**) summaries (n = 5 for each) of responses (top) to 1 μM PGI_2_ and forces of PE-evoked contractions (pre-force; bottom) in control L-NAME-treated TP^−/−^ vessels or those additionally with the EP3 antagonist L798106 (1 μM; +L) or the EP1 antagonist SC19220 (10 μM; +SC). (**b**) representative traces with summarized values showing the control response to PGI_2_ in endothelium-denuded TP^−/−^ vessels [TP^−/−^/EC (−)] or that with L798106 (+L). *P < 0.05. (**c**) summaries (n = 5 for each) of responses (top) to 0.1 μM PGE_2_ and forces of PE-evoked contractions (bottom) as in (**a**). **P < 0.01 vs. control value of TP^−/−^ vessels. In (**a–c**), *P < 0.05 or **P < 0.01 vs. control value of TP^−/−^ vessels. Data are expressed as mean ± SEM. (**d**) representative traces showing the response to PGE_2_ in L-NAME-treated TP^−/−^ vessels (TP^−/−^) or that obtained with L798106 (+L).

Next, we determined whether PGI_2_-evoked contractile response in TP^−/−^ vessels was masked by a dilator effect of the agonist. To this end, L-NAME-treated or endothelium-denuded abdominal aorta were precontracted with phenylephrine (PE; 2 μM), under which the vasoconstrictor response to an agonist is more readily detectable compared to baseline conditions^28^. Under either condition, PGI_2_ (1 μM) evoked an increase of force on PE-induced contraction, which was however reversed by the EP3 antagonist L798106 (1 μM) into relaxation (Fig. 3a,b). In contrast, the EP1 antagonist SC19220 (10 μM) had no effect (Fig. 3a). Also, L798106, but not SC19220 inhibited a similar response evoked by PGE_2_ (0.1 μM), although no relaxation was observed (Fig. 3c,d). Meanwhile, forces of PE-evoked contractions were found to be comparable among vessel groups that had been treated with L-NAME (Fig. 3a,c bottom panels).

### Effect of EP3 antagonism on ACh-evoked contraction in TP^−/−^ or TP-inhibited abdominal aortas

In the mouse abdominal aorta, the muscarinic agonist acetylcholine (ACh) activates endothelial COX to mainly produce PGI_2_ and evoke contraction under NOS-inhibited conditions^8^,^28^,^35^. Therefore, responses evoked by the maximal concentration of ACh were examined in L-NAME-treated TP^−/−^ or TP-inhibited abdominal aortas^8^,^13^,^14^,^15^.

**Figure 4:**
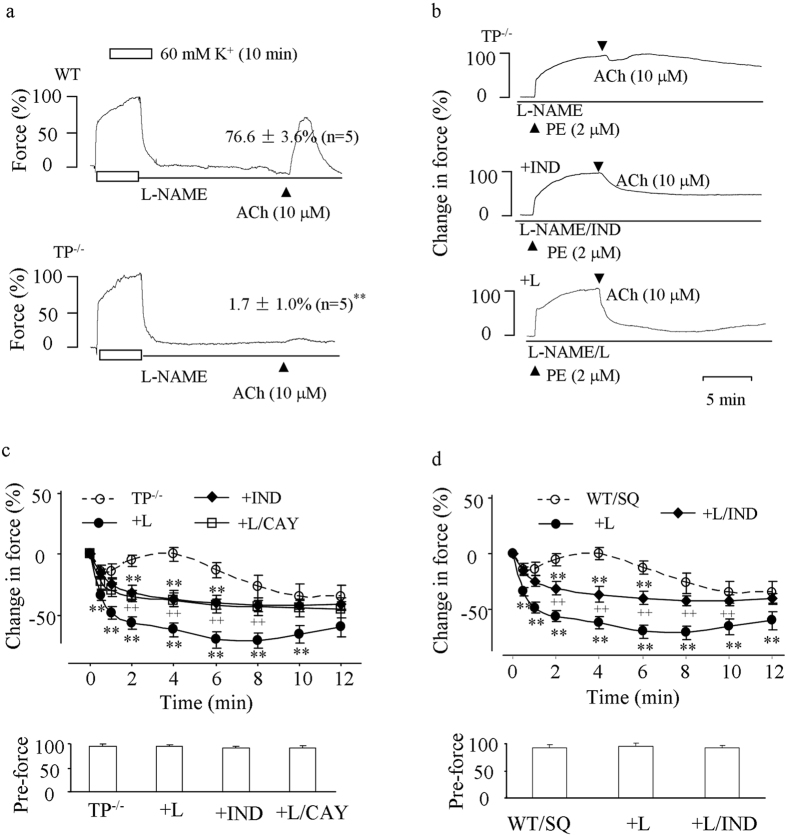
Responses to ACh in L-NAME-treated TP^−/−^ abdominal aortas or those of WT mice with TP inhibited. (**a**) representative traces with summarized values showing responses to ACh (10 μM) under baseline conditions in WT (top) and TP^−/−^ (bottom) vessels. *P < *0.01 vs. WT vessels (**b,c**) representative traces (**b**) and/or summaries of time-courses of responses to ACh (**c** top) along with forces of PE-evoked contractions (pre-force; **c** bottom) in precontracted TP^−/−^ vessels or those obtained with the non-selective COX inhibitor indomethacin (10 μM; +IND), with the EP3 antagonist L798106 (1 μM; +L) or with both L789106 and the IP antagonist CAY10441 (1 μM; +L/CAY). (**d**) time-courses of responses to ACh (top) and forces of PE-evoked contractions (pre-force; bottom) in precontracted WT vessels in the presence of the TP antagonist SQ29548 (WT/SQ) or in those additionally treated with L798106 (1 μM; +L) or both L798106 and indomethacin (+L/IND). In (**c** and **d**) **P < 0.01 vs. TP^−/−^ or WT/SQ; ^++^*P* < 0.01 vs. TP^−/−^/L or +L. Data were expressed as mean ± SEM (n = 5 for each).

As compared to that of WT controls, the contraction evoked by ACh (10 μM) in TP^−/−^ vessels was indeed mostly abolished; however, a minor contraction could still be produced (Fig. 4a). Moreover, in such-treated TP^−/−^ vessels precontracted with PE (2 μM), ACh evoked relaxation, which was blunted by a biphasic force sensitive to the non-selective COX inhibitor indomethacin. Interestingly, the EP3 antagonist L798106 also abolished the biphasic force, resulting in relaxation, which was to a greater extent than that obtained with indomethacin (Fig. 4b,c). In addition, such an enhancement of relaxation resulting from L798106 was removed by the IP antagonist CAY10441 (1 μM; Fig. 4c).

Likewise, in similar PE-precontracted WT vessels where the agonist usually evokes an increase of force^28^, treatment with the TP antagonist SQ29548 (10 μM) caused relaxation that was also blunted by a biphasic force in response to ACh. Again, the EP3 antagonist L798106 (1 μM) abolished the force, resulting in an enhanced relaxation that was reduced by indomethacin (Fig. 4d top). No significant difference was found among forces of PE-evoked contractions in TP^−/−^ or WT vessels (Fig. 4c,d bottom panels).

### Effect of TP^−/−^ on endothelial COX products and expressions of PG receptors

**Figure 5:**
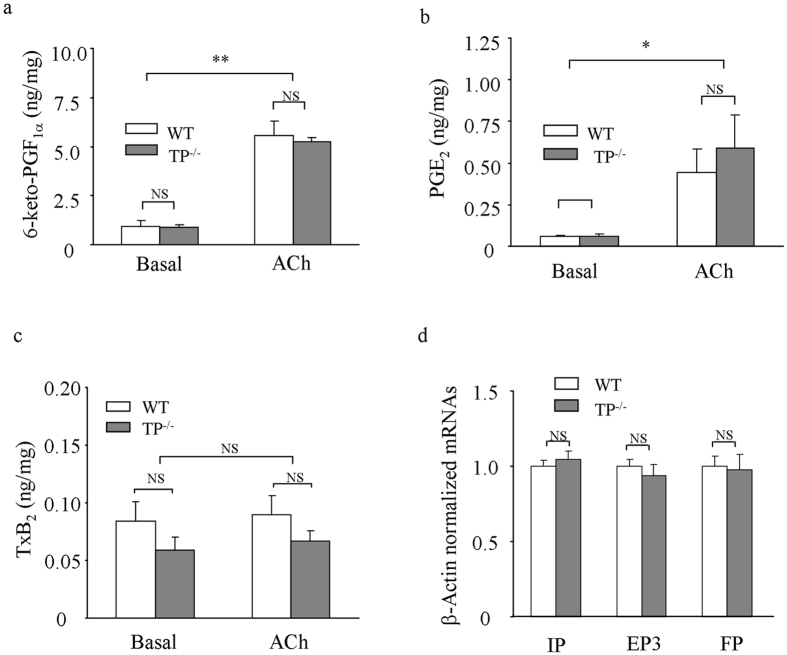
Effect of TP^−/−^ on COX products and IP, EP3 and FP mRNA levels. (**a–c**) summaries of the PGI_2_ metabolite 6-keto-PGF_1α_ (**a**), PGE_2_ (**b**), and the TxA_2_ metabolite TxB_2_ (**c**) in TP^−/−^ and WT aortas under the basal and ACh (10 μM)-stimulated conditions. (**d**) real-time PCR detection of IP, EP3 and FP mRNAs in TP^−/−^ and WT aortas. The level of mRNAs was normalized by that of β-actin with the average value of WT assumed as 1.0. *P < 0.05 and **P < 0.01; NS: not significant. Data are expressed as mean ± SEM (n = 6 for each).

The production of PGI_2_ and some other prostanoids evoked by ACh in TP^−/−^ and WT aortas was then examined. As shown in Fig. 5a, in WT and TP^−/−^ aortas ACh evoked an increase in the production of the PGI_2_ metabolite 6-keto-PGF_1α_, which was comparable between the two mouse strains. Also, an increase of PGE_2_ was obtained with ACh, although levels were ~10-fold lower than those of 6-keto-PGF_1α_ (Fig. 5b). No significant difference was found in amounts of PGE_2_ between TP^−/−^ and WT vessels (Fig. 5b). In contrast, the TxA_2_ metabolite TxB_2_ was not increased by ACh in vessels from either mouse strain (Fig. 5c), similar to results reported previously^34^,^36^.

The expressions of IP, EP3 and the PGF_2α_ receptor (FP) mRNAs were also determined. As shown by real-time PCR, the level of IP, EP3 or FP mRNAs normalized by that of β-actin in TP^−/−^ aortas was comparable with that of WT counterparts (Fig. 5d).

### Effect of EP3 antagonism on varied vasoconstrictor responses in WT vessels

**Figure 6:**
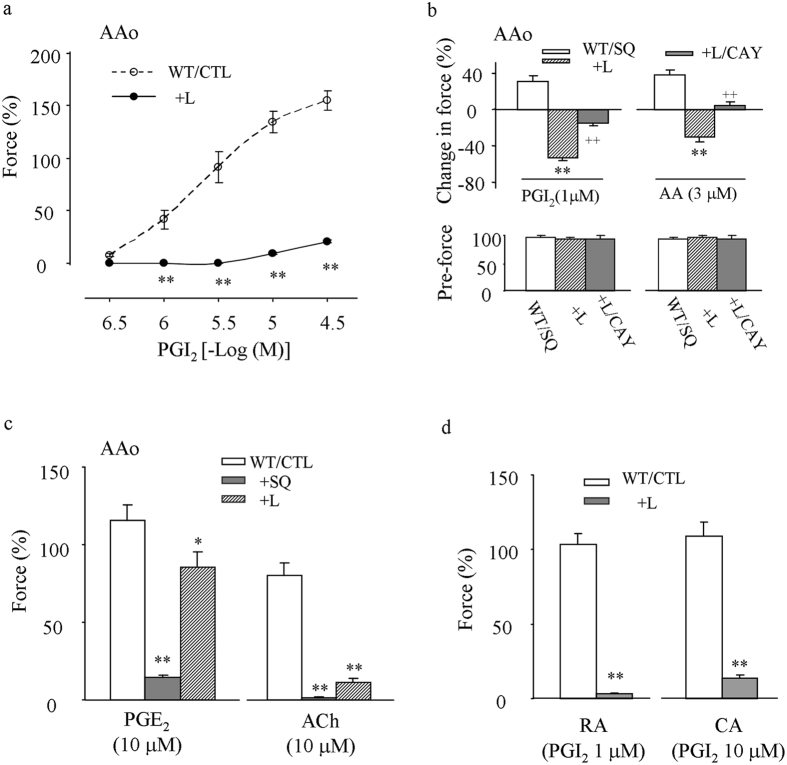
Effects of EP3 antagonism on vasoconstrictor responses in L-NAME-treated WT vessels. (**a**) control (CTL) responses evoked by PGI_2_ under baseline conditions in the abdominal aorta (AAo), and that obtained with the EP3 antagonist L798106 (1 μM; +L) (**b**) responses (top) to PGI_2_ (1 μM) or AA (3 μM) and forces of PE-evoked contractions (pre-force; bottom) in pre-contracted AAo treated with the TP antagonist SQ29548 (10 μM; WT/SQ) or those additionally with L798106 (1 μM; +SQ/L) or both L789106 and the IP antagonist CAY10441 (1 μM; +SQ/L/CAY). **or ^++^*P* < 0.01 vs. WT/SQ or +SQ/L, respectively. (**c**) effect of L789106 (1 μM; +L) or SQ29548 (10 μM; +SQ) on the contraction to PGE_2_ (10 μM; PGE_2_) or ACh (10 μM) in AAo. (**d**) effect of L789106 (1 μM; +L) on contraction to 1 or 10 μM PGI_2_ in carotid (CA) and renal arteries (CA), respectively. In (**a**,**c** and **d**) *P < 0.05, and **P < 0.01 vs. WT control (WT/CTL). Data are expressed as mean ± SEM (n = 5 for each).

The effect of EP3 antagonism was further determined in L-NAME-treated WT vessels. As shown in Fig. 6a, in WT abdominal aortas the EP3 antagonist L798106 (1 μM) diminished the contraction evoked by PGI_2_. Also, in such vessels precontracted with PE (2 μM), PGI_2_ (1 μM) or the COX substrate AA (3 μM), whose response is sensitive to TP antagonism under baseline conditions^35^, evoked an increase of force in the presence of the TP antagonist SQ29548 (10 μM) but a relaxation that was sensitive to the IP antagonist CAY10441 (1 μM) when both SQ29548 and L798106 were present (Fig. 6b). No significant difference was found among forces of PE-evoked contractions (Fig. 6b bottom).

Interestingly, in WT abdominal aortas, L798106, which drastically diminished the contraction evoked by ACh, only slightly reduced that evoked by a sub-maximal concentration of PGE_2_ (10 μM; Fig. 6c). However, this contraction to PGE_2_ was very sensitive to the TP antagonist SQ29548 (Fig. 6c). In addition, L798106 diminished the contraction evoked by 1 or 10 μM PGI_2_ in carotid and renal arteries (Fig. 6d).

## Discussion

In this study we show that in NOS-inhibited WT mouse abdominal aortas PGI_2_- or ACh (which activates endothelial COX to mainly produce PGI_2_) evokes contraction that is diminished in TP^−/−^ counterparts. More importantly, in TP^−/−^ vessels or TP-inhibited vessels of WT mice, antagonizing EP3 abolishes the remaining vasoconstrictor responses to these agonists, resulting in relaxations sensitive to IP and/or COX blockade. EP3 antagonism also diminishes the contraction evoked by PGI_2_ and/or ACh in NOS-inhibited WT abdominal aorta, carotid and renal arteries. These results not only demonstrate that TP contributes only partially to the contraction evoked by PGI_2_, but also suggest that EP3 has an important involvement in the response.

The deletion of TP in TP^−/−^ mice was confirmed by DNA sequencing, mRNA analyses and an elongation of bleeding time^37^. Indeed, abdominal aortas from these mice (which posses a normal MAP, as reported previously^38^) lost contraction in response to the TxA_2_ analogue and TP agonist U46619 even under NOS-inhibited conditions. Notably, in such vessels, not only the contraction to PGI_2_, but also that to PGF_2α_, PGE_2_, or PGD_2_ was diminished. In contrast, levels of IP, FP and EP3 mRNAs were similar between TP^−/−^ and WT vessels, arguing against that the above reduced PG responses resulted from altered expressions of receptors. Thus, TP, which appears able to be activated by all vasoactive prostanoids that are structurally similar^4^, mediates PGI_2_’s contractile activity. Due to practical reasons, we were unable to clearly detect IP, EP3, and FP at the protein level; however, our above idea concurs with results in WT mice and some other species obtained here or previously with pharmacological blockade^8^,^28^,^34^. At the same time, responses evoked by low concentrations (0.1–0.3 μM) of PGE_2_ in TP^−/−^ vessels reveal a functional role of EP3 unaffected by the TP antagonist used.

Interestingly, we further noted that antagonizing EP3 abolished the remaining contraction, resulting in relaxation in response to PGI_2_ in either NOS-inhibited or endothelium-denuded TP^−/−^ abdominal aortas. This suggests that EP3, which exists in medial smooth muscles in a manner similar to that of IP and TP, mediates PGI_2_’s contractile response, although its effect is largely offset by IP when TP is absent. In support of the idea, in NOS-inhibited, TP-antagonized WT vessels a relaxation sensitive to IP antagonism was also evoked by PGI_2_ following EP3 blockade. Moreover, after EP3 antagonism the contraction to PGI_2_ was minimal. This suggests that the part of EP3-mediated activity could be only slightly smaller than that of TP, which alone could also be largely masked by IP-mediated effect. Therefore, the robust contractile response to PGI_2_ in WT vessels reflects activities from both TP and EP3 overcoming the effect of concomitantly activated IP. In contrast, EP1 (another vasoconstrictor PGE_2_ receptor), EP2 and EP4 (dilator PGE_2_ receptors) do not appear to have a role in the vessels studied, as suggested by the lack of effect caused by antagonism or the absence of relaxation to PGE_2_ in TP^−/−^ vessels even after EP3 is antagonized^39^,^40^.

Also, the above effects of TP^−/−^, TP and/or EP3 antagonism under NOS-inhibited conditions were similarly obtained in responses evoked by ACh or AA, which stimulates endothelial COX to mainly produce PGI_2_^28^,^35^. As seen from EIA measurements, the profile of COX-derived products in aortas was unaltered by TP^−/−^. Thus, the mechanism for the contraction evoked by endothelial COX metabolites produced *in situ* is similar to that of PGI_2_. Due to an endothelium-derived hyperpolarizing factor (EDHF)-mediated relaxation concomitantly activated^28^,^41^, the effect of EP3 antagonism on the response evoked by ACh in NOS-inhibited, TP^−/−^ or TP-inhibited vessels was reflected by abolition of the contractile activity blunting EDHF-mediated relaxation and a relaxation that is sensitive to IP or COX blockade, but adds to that of EDHF. One must note that the EDHF activity in the vessel does not originate from non-COX AA metabolites, as we put forward previously^35^. Indeed, this point is also supported here by the lack of IP-independent relaxation to AA after TP and EP3 were both antagonized.

Previously, the contractile role of EP3 in PGE_2_-evoked vasoresponse was established in vessels of mice as well as those of humans^32^,^42^. In the present study, our results further suggest an intimate link between EP3 and the contractile activity evoked by PGI_2_. Notably, PGD_2_ might also act on EP3 to mediate a minor contraction, as revealed by functional studies of its response in TP^−/−^ vessels. These results could again be possibly due to a structural similarity among PGs. In support of this, iloprost, a PGI_2_ analog, also activates EP3^43^. Moreover, EP3 antagonism exerts a greater inhibitory effect on PGI_2_-evoked contraction than on that of PGE_2_ (whose response via EP3 peaks at 0.3 μM, as seen from its response in TP^−/−^ vessels). This implies not only that the EP3 antagonist used has limited if any, unintended effects on TP, but also that PGI_2_, although it might have a lower potency, is more effective on EP3 than PGE_2_, underscoring the importance of PGI_2_ in EP3-mediated vasoconstrictor activities. Besides, the effects of its antagonism among WT vessels studied further indicate that the involvement of EP3 in PGI_2_’s vasoconstrictor activity is not limited to any specific vascular bed.

Therefore, our above results make important amendments to previous proposals on the mechanisms of PGI_2_ or endothelial COX metabolites (which consist mainly of PGI_2_) in mediating vasoconstrictor responses^20^,^44^. It should be noted that the contraction to PGI_2_ only exists in vessels with limited expression of IP^44^. This is also true in the abdominal aorta examined here where we previously showed that IP expression is lower than in mesenteric arteries (where 0.3 μM PGI_2_ almost completely relaxes 2 μM PE-evoked contraction)^28^. A reason for this could be that PGI_2_ (the prototype IP agonist) is more potent on IP than TP and/or EP3, leading to PGI_2_ being more likely to evoke relaxation than to cause contraction. Indeed, this idea explains why PGI_2_ has been recognized as a potent vasodilator in many vascular beds and used clinically as an effective therapy for pulmonary hypertension or peripheral arterial diseases^2^,^6^,^45^,^46^.

On the other hand, it must also be emphasized that the minimal concentration of PGI_2_ required to initiate vasoconstrictor activity could be 0.003–0.03 μM (under precontracted conditions), far below the amount (1 ng/mg 6-keto-PGF_1α_ can be translated into 2.7 μmol PGI_2_ per kg of vessel) released by agonists, such as ACh, or similar to that of it (PGI_2_) to evoke relaxation in vessels, such as mouse mesenteric arteries even after TP is antagonized^28^,^30^,^44^. Also, PGI_2_-mediated contraction or endothelial COX-derived vasoconstrictor activity has been found in many vessels across species (including those of humans), of which some are small or resistance arteries^23^,^24^,^29^,^34^,^47^. Moreover, PGI_2_’s contractile activity exists in vessels that show a dilator response to the agonist^27^,^32^. As a result, although PGI_2_ may cause a hypotensive effect in general, concurrent activities via TP and/or EP3 can negate some of its beneficial effects via IP, especially on local vascular pathology under disease conditions^22^,^26^,^36^. For this reason, antagonizing TP and/or EP3 might be needed for an optimal therapeutic effect obtained with PGI_2_ or its analogues under clinical conditions.

In contrast to our findings, EP1 antagonism has also been suggested to diminish PGI_2_-evoked contraction^48^. However, the EP1 antagonist used was also a partial antagonist of TP, which was deleted in the vessels we studied^49^, not to mention the variation that might exist among species or vascular beds. Also, the COX inhibitor indomethacin may cause off-target effects^50^,^51^; however, this agent has been shown not to alter ACh responses in similar vessels of COX-1^−/−^ mice^28^. Indeed, IP blockade inhibited the relaxation in a manner similar to that of indomethacin. Thus, the effect of indomethacin noted here can be considered to result mainly from COX inhibition. However, the precise structural properties responsible for different PGs to activate the same receptor or for one PG to act on different receptors still require further investigation. Also, reasons for one PG to evoke contraction mainly through receptors other than its own, e.g. FP of PGF_2α_ need to be resolved, given that contractions evoked by endothelial COX metabolites can result from non-PGI_2_ products, including PGF_2α_^19^,^20^,^34^,^52^,^53^.

In summary, our results demonstrate that TP, which appears able to be activated by all vasoactive prostanoids, only partially mediates PGI_2_’s vasoconstrictor activity. Interestingly, our data further suggest that PGI_2_ also effectively activates EP3, whose activity along with that of TP can overcome the dilator effect of concomitantly activated IP to produce a robust vasoconstrictor response, and hence imply a novel mechanism for endothelial COX metabolites (which consist mainly of PGI_2_) in regulating vascular functions.

## Material and Methods

### Chemicals and solution

L-NAME, ACh, PE, AA, and the non-selective COX inhibitor indomethacin were purchased from Sigma (St Louis, MO, USA). The TP agonist U46619, PGI_2_, PGF_2α_, PGE_2_, and PGD_2_, the TP antagonist SQ29548, the IP antagonist CAY10441, the EP3 antagonist L798106, and the EP1 antagonist, SC19220 were bought from Cayman Chemical (Ann Arbor, MI, USA). The composition of physiological salt solution (PSS; pH 7.4 with 95%O_2_–5% CO_2_) was as follows (in mM): NaCl 123, KCl 4.7, NaHCO_3_ 15.5, KH_2_PO_4_ 1.2, MgCl_2_ 1.2, CaCl_2_ 1.25, and D-glucose 11.5. The 60 mM K^+^ -PSS (K^+^) was prepared by replacing an equal molar of NaCl with KCl.

L-NAME, PE, AA, and ACh were dissolved in distilled water (purged with N_2_ for dissolving AA), while PGI_2_ was dissolved in carbonate buffer (50 mM, pH 10.0). PGF_2α_, PGE_2_, PGD_2_, CAY10441, SQ29548, L798106, and indomethacin were dissolved in dimethyl sulfoxide (DMSO). The final ratio of a solvent (distilled water, carbonate buffer, or DMSO) to working PSS was 0.5/1,000, which doesn’t alter the final pH value of the working buffer (pH 7.4). The concentration of an inhibitor or antagonist used was based on previous reports, which would selectively inhibit the effect of its intended target^27^,^54^,^55^.

### Animals and tissue preparation

All procedures were in conformance with the Guide for the Care and Use of Laboratory Animals published by the US National Institutes of Health (NIH Publication No. 85–23, revised 1996), and approved by The Institutional Animal Research and Use Committee of Shantou University.

The breeder male and female TP^−/−^ mice (C57BL/5 background) were custom produced by Viewsolid Biotech (Beijing, China), using transcription activator-like effector nuclease technology that targets the TP gene to result in deletion of a 22 bp DNA fragment, 18 bp after the start codon in exon 3 of the TP locus^56^. WT mice (C57BL/6) were purchased from Vital River (Beijing, China). Male WT mice or TP^−/−^ progenies of 8–12 wk of age were used for experiments. Mice were killed by CO_2_ inhalation. For *in vitro* functional and biochemical analyses, aortas, carotid and/or renal arteries were isolated and dissected free of adherent tissues with the help of a binocular microscope.

### DNA sequencing

The gene mutation in TP^−/−^ mice was verified by sequencing PCR products (the sense and anti-sense PCR primers were 5′-GAA AGG GTA TTT TGT TCC TGA GGC-3′ and 5′-GCT ACC CCC ATG AAG TAG CAC AGG-3′, respectively) of DNA isolated from tail biopsies and performed by Sangon Biotech (Shanghai, China).

### RT-PCR and real-time PCR

The preparation of total RNA from whole sections of mouse aortas and RT reactions were performed as described elsewhere previously^28^. First-strand cDNA was synthesized using total RNA (250 ng) and oligo(dT)15 primers (TaKaRa; Dalian, China).

TP mRNA transcripts were detected with RT-PCR. Primers for TP were 5′-CTG GGG GCC TGC TTT CGC CCG G-3′ (sense; using the deleted fragment) and 5′–GTC AGG AAG CAC CAA GAG CC-3′ (antisense), while those for β-actin (internal control) were as described previously^28^. The expected sizes of the RT-PCR products were 530 bp for TP and 300 bp for β-actin.

Expressions of IP, EP3 or FP mRNAs were analyzed by real-time PCR. Primers for EP3 or FP were as follows: 5′-CAG AAT CAC CAC GGA GAC G-3′ (EP3 sense) and 5′-TGC ATT GCT CAA CCG ACA T-3′ (EP3 antisense), and 5′-TCC TTG GAC ACC GAG ATT AT-3′ (FP sense) and 5′–GCA ACG ACT GGC AAG TTT AT-3′ (FP antisense). Those for IP and β-actin (internal control) were described previously^28^. Real-time PCR was performed using a SYBR PrimScript RT-PCR kit (Thermo Scientific, Carlsbad, CA, USA).

### Blood pressure measurement

In some experiments, blood pressure in mice (body weight of 26–30 g) was measured using a computerized noninvasive blood pressure system (Kent Scientific Corporation, Torrington, CT, USA). Mice were accustomed to tail-cuff blood pressure measurements for 3 consecutive days, and then blood pressure was measured on the 4^th^ day. MAP taken from the averaged value of three measurements was used for analysis.

### Tail bleeding time assay

To evaluate *in vivo* bleeding time, WT and TP^−/−^ tails (age, tail size and length matched) were cut 2 mm from the tips, and wounds were then gently wiped with sterilized filter paper every 30 s, until no more blood was visible. The bleeding time was calculated from the ending of cutting to the time when no more blood would be seen on paper.

### Analysis of vascular function

Abdominal sections of aortas and main stems of carotid or renal arteries were cut into 1 mm rings. Analysis of vascular function was performed with isometric force measurement as described elsewhere previously^28^,^30^. For some experiments, the endothelium was denuded by rotating vessel rings around two wires with passive tension kept at 100 mg (endothelial removal was confirmed by absence of relaxation to 10 μM ACh at the end of experiment).

To remove the influence of endothelial NO, vessels were treated with the NOS inhibitor L-NAME (1 mM), under which the response of arteries appears similar to that of eNOS^−/−^ mice^14^. Inhibitors or solvents were added 30 min before the vessel was contracted with an agonist, and was kept in the solution throughout the experiment. The response elicited by an agonist under baseline conditions was expressed relative to the contraction evoked by 60 mM K^+^, while that during the contraction evoked by PE (2 μM) was expressed as a change of force relative to the value before the application of the agent.

### Assay of COX-derived metabolites

Measurement of the PGI_2_ metabolite 6-keto-PGF_1α_, the TxA_2_ metabolite TxB_2_, or PGE_2_ was performed by EIA^28^,^36^. Briefly, after being rinsed of blood components, whole sections of aortas were incubated with PSS at 37 °C for 30 min, followed by exposure to PSS (300 μl) and ACh (10 μM) in 300 μl PSS (37 °C) for 15 min each. Thereafter, vessels were taken out, and 1, 10, or 100 μl of reaction solutions was used for 6-keto-PGF_1α_, PGE_2_, or TxB_2_ measurements, respectively (2 replicates for each single measurement), using protocols according to instructions of the manufacturer. The amount of 6-keto-PGF_1α_, TxB_2_, or PGE_2_ was expressed in ng per mg of wet tissue.

### Data analysis

Values were expressed as means ± SEM from n numbers or pools of vessels from different animals. The normality of data sets with n of 5 or more was confirmed using the Kolmogorov-Smirnov test. Thereafter, statistical analyses were performed with a Student’s t-test (unpaired) or ANOVA (1-way or 2-way), followed by Bonferroni’s or Dunnett’s post-hoc test. For some data sets with undeterminable normality (n = 3), the Mann-Whitney U test was used. *P* < 0.05 was considered to be statistically significant.

## Additional Information

**How to cite this article**: Li, Z. *et al*. Role of E-type prostaglandin receptor EP3 in the vasoconstrictor activity evoked by prostacyclin in thromboxane-prostanoid receptor deficient mice. *Sci. Rep.* **7**, 42167; doi: 10.1038/srep42167 (2017).

**Publisher's note:** Springer Nature remains neutral with regard to jurisdictional claims in published maps and institutional affiliations.
